# Elements of Histology

**Published:** 1884-01

**Authors:** 


**Elements of Histology.** By E. KLEIN, M. D., F. R. S., Joint Lecturer on General Anatomy and Physiology in the Medical School of St. Bartholomew's Hospital, London. Illustrated with 181 engravings. Philadelphia: Henry C. Lea's Son & Co. 1883.

In the words of the preface: "This work is intended as a manual for medical students. The original plan was to embody in it only such matter as was absolutely required for the first year's student in the medical school, but this plan was not carried out, since the book would then have been, in some measure, incomplete as a manual of histology. The work contains a good deal that will be acceptable to the advanced student as well as the beginner." This little work is one of a set of five manuals for medical students. It is a small 12mo. of 350 pages. Little works of this character and scope are exceedingly useful as giving the medical man, not a specialist, quite a complete understanding of such subjects as histology, pathology, etc. And they possess this additional advantage, that they will be read where works of a more comprehensive and exhaustive nature are laid aside for only an occasional reference. We recommend the little book in the same way as Green's Manual of Pathology, of which we have always had a highly favorable opinion.

